# CD2v Interacts with Adaptor Protein AP-1 during African Swine Fever Infection

**DOI:** 10.1371/journal.pone.0123714

**Published:** 2015-04-27

**Authors:** Daniel Pérez-Núñez, Eduardo García-Urdiales, Marta Martínez-Bonet, María L. Nogal, Susana Barroso, Yolanda Revilla, Ricardo Madrid

**Affiliations:** 1 Virology Department, Centro Biología Molecular Severo Ochoa, CSIC-UAM, Madrid, Spain; 2 Molecular Design Group, School of Biochemistry & Immunology, Trinity Biomedical Sciences Institute, Trinity College Dublin, Pearse Street, Dublin, Ireland; 3 Hospital Gregorio Marañón_,_ Madrid, Spain; Lisbon University, PORTUGAL

## Abstract

African swine fever virus (ASFV) CD2v protein is believed to be involved in virulence enhancement, viral hemadsorption, and pathogenesis, although the molecular mechanisms of the function of this viral protein are still not fully understood. Here we describe that CD2v localized around viral factories during ASFV infection, suggesting a role in the generation and/or dynamics of these viral structures and hence in disturbing cellular traffic. We show that CD2v targeted the regulatory trans-Golgi network (TGN) protein complex AP-1, a key element in cellular traffic. This interaction was disrupted by brefeldin A even though the location of CD2v around the viral factory remained unchanged. CD2v-AP-1 binding was independent of CD2v glycosylation and occurred on the carboxy-terminal part of CD2v, where a canonical di-Leu motif previously reported to mediate AP-1 binding in eukaryotic cells, was identified. This motif was shown to be functionally interchangeable with the di-Leu motif present in HIV-Nef protein in an AP-1 binding assay. However, we demonstrated that it was not involved either in CD2v cellular distribution or in CD2v-AP-1 binding. Taken together, these findings shed light on CD2v function during ASFV infection by identifying AP-1 as a cellular factor targeted by CD2v and hence elucidate the cellular pathways used by the virus to enhance infectivity.

## Introduction

African swine fever (ASF) is a highly lethal and economically important disease of domestic pigs for which there is no control strategy other than animal quarantine and slaughter. Classified as a notifiable disease by the World Organization for Animal Health (OIE), ASF causes major economic losses to the pig industry in affected countries. Despite the high risk represented by the recent outbreak in the Caucasus in 2007, its subsequent propagation throughout Russia and potential dissemination to neighboring countries [1], to date, no specific protection or vaccine against ASF is available.

The causative agent of ASF is African swine fever virus (ASFV), a large enveloped, icosahedral, double-stranded DNA virus, the only member of the *Asfarviridae* family [2], with an approximately 170 kpb genome encoding at least 150 proteins, including proteins able to modulate virus-host interactions [3–7]. ASFV infects cells of the mononuclear-phagocytic system, including highly differentiated fixed tissue macrophages [8,9] that enter the cell by mechanisms involving macropinocytosis [10]. In domestic pigs, ASF occurs in several different forms ranging from highly lethal to subclinical, in which the contribution of viral and host factors remains poorly understood.

CD2v is an ASFV protein that resembles the T-lymphocyte surface adhesion receptor CD2. It contains an extracellular N-terminal (Nt) domain composed of two immunoglobulin-like domains, while the cytosolic C-terminal domain of CD2v (CD2v-Ct) shares no obvious amino acid sequence with the cellular CD2 cytoplasmic domain [11]. During infection, CD2v is assumed to be cleaved into a Nt glycosylated and a Ct non-glycosylated form, but both coexist in the infected cell with the full length protein [12]. CD2v expression is required for the hemadsorption phenomenon observed in ASFV infected cells, which is presumably caused by its extracellular domain [11]. This may provide a mechanism for viral spread, since in pigs infected with a recombinant ASFV Malawi strain lacking the CD2v protein (Malawi Δ8DR), both dissemination to lymph nodes and the onset of disease were delayed, although the mortality rates were similar [13]. CD2v appears to be truncated in attenuated strains, suggesting the involvement of the protein in ASFV pathogenesis [14]. CD2v has also been reported to be involved in the inhibition of lymphocyte proliferation [13] and in replication in the tick vector [15]. On the other hand, the ectopically expressed CD2v-Ct domain appears to be responsible for its Golgi location around the viral factory during infection [12] and for binding to the actin-binding cellular protein SH3P7 [16]. Hence, CD2v appears to be involved in both the virulence and in the pathogenicity of ASFV *in vivo* [3,13,14] although the roles it plays during infection and its contribution to virulence and pathogenicity are not yet well understood.

Adaptor protein 1 (AP-1) is a cytosolic heterotetramer involved in the transport of protein cargo from the trans-Golgi network (TGN) to endosomes [17]. AP-1 recruits clathrin to form clathrin-coated vesicles and plays a pivotal role in selecting the cargo by recognizing sorting signals in the cytoplasmic tail of integral membrane proteins. To date, two sorting signals have been identified and well characterized: the tyrosine (YXXΦ) and the di-leucine ([D/E]XXXL[L/I) motifs [18] selectively recognized by AP-1, which directly binds them. Recruitment of AP-1 to the TGN membrane is regulated by the small GTPase ADP-ribosylation factor 1 (Arf1) which, in turn, is regulated by Golgi-associated GTPase-activating proteins (GAPs) and guanine nucleotide exchange factors (GEFs) [19]. Some of these GEFs families (such as GBF1, BIG1/BIG2) are sensitive to brefeldin A (BFA) [20]. BFA inhibition of GEFs triggers the release of Arf1 from the Golgi membranes and hence of AP-1 [21].

Binding to AP-1 might involve reorganization of Golgi and cellular traffic events during ASFV infection, presumably with the aim of facilitating viral replication, encapsulation and/or egress; this kind of viral strategy appears to be a common mechanism of increasing virulence and disease progression in other viruses. For example, HIV-Nef binds to AP-1 through a well characterized di-Leucine (di-Leu) motif [22,23]. This binding leads to the stabilization of AP-1 on the membranes [24] and results in alteration of the endocytic pathway, correlating with an increase in virulence [25]. Nef also induces the binding of AP-1 to the MHC-I cytoplasmic tail, which is believed to play a role in MHC-I down-regulation and cross-presentation [26,27]. On the other hand, E6 protein from bovine papillomavirus type 1 (BPV-1) also binds to AP-1 [28], whereas the interaction between AP-1 and glycosylated proteins of herpes simplex virus is involved in viral spread [29,30].

Here for the first time, we describe CD2v binding to AP-1 and localizing around the viral factory during ASFV infection. The binding was sensitive to BFA, and, as a consequence, AP-1 was released from the TGN membrane. We showed that, in ASFV infected cells, AP-1 was dispersed into the cytoplasm, whereas CD2v remained attached to the membrane around the viral factory. Sequence analysis of CD2v with the ELM resource [31] identified a di-Leu motif predicted to mediate binding to AP-1. We demonstrate that this di-Leu motif was indeed functional in the HIV protein Nef, but, surprisingly, we found that the motif was not involved either in the colocalization or in the interaction between CD2v and AP-1. Finally, we were able to demonstrate that a region of the cytoplasmic tail of CD2v that does not contain the di-Leu motif, was able to interact with AP-1 in a pull-down assay, indicating that this region might harbor an as yet uncharacterized AP-1 binding motif.

Together, these results demonstrate a role for CD2v in AP-1 binding and location that could have direct consequences for traffic remodeling and virus infectivity, and target a new motif different from the canonical di-Leu as being responsible for the interaction between the viral protein and AP-1.

## Materials and Methods

### Cell culture, viruses and infections

COS-7 (COS) and Vero cells (African green monkey kidney) were obtained from the American Type Culture Collection (ATCC) and grown in Dulbecco's Modified Eagle's Medium (DMEM) supplemented with with 2 mM L-glutamine, 100 U/ml gentamicin, non-essential amino acids and 5% fetal bovine serum (FBS) (Invitrogen Life Technologies). Swine alveolar macrophages were prepared by bronchoalveolar lavage as described in [32] and grown in DMEM supplemented with 2 mM L-glutamine, 100 U/ml gentamicin, non-essential amino acids and 10% porcine serum. Cells were grown at 37°C in a 7% CO_2_ atmosphere saturated with water vapor.

The ASFV isolates E70, NH/P68 (NHV) and Ba71V were propagated and titrated by plaque assay on COS cells, as previously described [32,33]. Briefly, subconfluent Vero (Ba71v) and COS (E70 and NHV) cells were cultivated in roller bottles and infected with ASFV at a multiplicity of infection (MOI) of 0.5 in DMEM 2% FBS. At 72 h post infection (hpi), the cells were recovered and centrifuged at 3,000 rpm for 15 min. The cell pellet was discarded. The supernatant containing the viruses was clarified at 14,000 rpm for 6 h at 4°C and the purified infectious virus was resuspended in medium and stored at −80°C. ASFV viral adsorption to cells was performed at 37°C for 90 min to allow infection at 37°C until the hpi indicated.

### Antibodies and reagents

Monoclonal mouse anti-γ-Adaptin antibody (α-AP-1) was purchased from Sigma-Aldrich (A4200); monoclonal mouse anti-P72 (clone 17LD3) was a kind gift from Ingenasa; monoclonal anti-Vimentin (clone V9, sc-6260) and monoclonal mouse anti-GST (clone B14, sc-138) were purchased from Santa Cruz Biotechnology. Polyclonal rabbit anti-CD2 was previously generated by Fernando Almazán [34]. Topro3, DAPI, Phalloidin Alexa-647, anti-mouse Alexa Fluor-488, anti-mouse Alexa Fluor-555 and anti-rabbit Alexa Fluor-555 were purchased from Invitrogen, and anti-rabbit and anti-mouse immunoglobulin G coupled to peroxidase from Amersham Biosciences.

Tunicamicin (5μg/ml) was purchased from *Boehringer* Mannheim. Brefeldin A (1 μg/ml) was purchased from Sigma. Cycloheximide (100 μg/ml) was purchased from Sigma.

### Plasmid constructs

Overlap PCR was used to insert the *full-length* EP402R gene (coding for CD2v) or the Ct fragment including the predicted trans membrane domain (122-402aa) into pEGFP-N1 (Promega) and the *full-length* EP402R into pcDNA-HA (see below), adding an *Eco*RI site and Kozac sequence to the 5′ end and a *Bam*HI site to the 3′ end of EP402R or both BamHI sites at the 5’ and 3’ end of the Ct fragment. The fragments were amplified by PCR from a lysate of cells infected by ASFV E70 using the following primers: 5’-AGCGCGAATTCGCCACCATGATAATAATAGTTATTTTTTTAATGTG-3’ (or 5’- ACGCTGGATCCGCCACCATGATTACATATAATTGTACTAATTTTTTAATAACATG-3’ in the case of CD2v-Ct) and 5’-TGCGCGGATCCAGAATAATTCTATCTACATGAATAAGCG-3’ (CD2v-GFP and HA-CD2v). pcDNA-HA was made by introducing HA epitope to pcDNA-3 (Invitrogen) previously digested by *Bam*HI and *Not*I. The oligonucleotides 5’-GATCCTACCCATACGACGTCCCAGACTACGCTTAAGC-3 and 5’- CCGGCGAATTCGCATCAGACCCTGCAGCATACCCATC-3’ were incubated at 95°C for 2 minutes and then slowly cooled to 50°C to form the HA epitope, which was then ligated to pcDNA-3. L394A and I395A punctual mutation (LLAA) from wild-type versions of CD2v-GFP and HA-CD2v were generated using the QuikChange II XL Site-Directed Mutagenesis Kit (Agilent) and the following primers: 5’-CATTATCTACACAAAATATTTCGGCTGCTCATGTAGATAGAATTAT-3’ and 5’- ATAATTCTATCTACATGAGCAGCCGAAATATTTTGTGTAGATAATG-3’.

Overlap PCR was used to insert Ct fragment EP402R gene (230-402aa), into pGEX 4T-1 (Pharmacia) adding a *Bam*HI site to the 5′ end and a *Not*I site to the 3′ end. The fragment was amplified by PCR from a lysate of cells infected by ASFV E70 using the following primers: 5’-AGCGCGGATCCATACGAAGAAAAAGAAAAAAACATG-3’ and 5’-TGCGCGCGGCCGCTTAAATAATTCTATCTACATGAATAAGCG-3’ to generate GST-CD2v. L394A and I395A punctual mutation (LLAA) from wild-type versions of GST-CD2v was obtained as explained above. L394A and I395A punctual mutation (LLAA) from wild-type versions of GST-CD2v was obtained as explained above. Overlap PCR was used to insert the Ct “short” fragment (CTS) (230–304 aa) into pGEX 4T-1 adding a *Bam*HI site to the 5′ end and a *Xho*I site to the 3′ end, using the following primers: 5’- AGCGCGGATCCATACGAAGAAAAAGAAAGAAACATGTTG-3’ and 5’- TCGAATCTCGAGTTATTTAGGTAGGGGAAATGGGTTG-3’. For the cloning of the 374–402 aa fragment of the Ct (CT2), the following oligonucleotides comprising the entire sequence of the fragment were synthesized by optimizing the nucleotide sequence for its expression in prokaryotic system: 5’-GATCCCCGCTGCCGAGCATTCCGCTGCTGCCCAATATCCCGCCATTATCTACACAAAATATTTCGCTTATTCATGTAGATAGAATTATT**TAA**C-3’ and 5’-’TCGAG **TTA**AATAATTCTATCTACATGAATAAGCGAAATATTTTGTGTAGATAATGGCGGGATATTGGGCAGCAGCGGAATGCTCGGCAGCGGG-3’, cloned in frame into pGEX-4T1 digested by BamHI/XhoI. The 4X tandem CT2 fragment was cloned by serial restriction and ligation steps using the BamHI/XhoI restriction sites placed at the 3’ end of the double stranded synthetic oligonucleotides described above.

pGEX-Nef (GST-Nef), was generously donated by S. Benichou (Institut Cochin, Paris, France). pGEX-Nef-LLAA was obtained using the following primers: 5´- GGAGAGAACACCAGCGCGGCACACCCTGTGAGCTGC-3´and 5´-CAGGCTCACAGGGTGTGCCGCGCTGGTGTTCTCTCC-3´. pGEX-Nef-LLCD2v (*Nef-di-Leu-CD2v*) was obtained by introducing the following punctual mutations: E160Q, T162I and L165I using the following primers: 5´GTAGAAGAGGCCAATAAAGGACAGAACATCAGCTTGATACACCCTGTGAGCCTGCATG-3´and 5´-CATGCAGGCTCACA GGGTGTATCAAGCTGATGTTCTGTCCTTTATTGGCCTCTTCTAC-3´.

### Transfection assays

COS cells were transfected with 1 μg of specific expression plasmids per 10^6^ cells using the *LipofectAMINE Plus Reagent* (Invitrogen) according to the manufacturer's instructions and mixing in *Opti-MEM* (Invitrogen). Cells were incubated at 37°C for 4 h in serum free medium, washed and incubated at 37°C for 24 h.

### Co-Immunoprecipitation assay

Cells were MOCK-infected or infected with ASFV E70 (MOI = 3). The infected cells were either treated or not with tunicamycin (*Boehringer* Mannheim) at 5 μg/ml, which was added after the virus adsorption. At 16 hpi cells were collected and lysed (25 mM Tris-HCl [pH7.5], 150 mM NaCl, 2 mM EDTA, 10 mM MgCl, 0.5% glycerol, protease inhibitors [0.8 μg/ml aprotinin, 0.8 μg/ml pepstatin and 0.8 μg/ml leupeptin], 1% Triton x-100). The extracts were incubated with a specific antibody against AP-1 (Anti-γ-Adaptin, Sigma-Aldrich) at a final concentration of 1.2 μg/ml overnight at 4°C. Protein G-Sepharose beads (Sigma-Aldrich) were added, incubated for 3 h at 4°C, and centrifuged. The beads were washed three times with wash buffer (25 mM Tris-HCl [pH 7.5], 150 mM NaCl, 2 mM EDTA). The immunoprecipitates were mixed with SDS loading buffer and analyzed by 10% SDS-PAGE, followed by Western blotting.

### GST pull-down assay

The Ct domain versions of CD2v or the wild-type or mutated Nef proteins fused to glutathione-*S*-transferase (GST) were produced in *Escherichia coli* by IPTG induction. COS cells (10^6^) were lysed in 50 mM Tris-HCl (pH 8), 5 mM EDTA, 150 mM NaCl, protease inhibitors (0.8 μg/ml aprotinin, 0.8 μg/ml pepstatin and 0.8 μg/ml leupeptin) and phosphatase inhibitors (PhosSTOP, Roche) and 1% Triton X-100. The cytoplasmic lysates were incubated overnight at 4°C with GST or GST-Nef or GST-CD2v proteins, immobilized on glutathione-sepharose beads (Amersham Biosciences). Beads were washed four times in lysis buffer; the bound cellular proteins were analyzed by Western blotting.

### Western blot analysis

Samples from co-immunoprecipitation or GST-pull-down assays were fractionated by SDS-PAGE and electrophoretically transferred to an Immobilon extra membrane (Amersham) and the separated proteins reacted with specific primary antibodies. The antibodies used were anti-γ-adaptin (AP-1) (dilution 1:1000), anti-CD2v (dilution 1:5000), anti-GST (dilution 1:1000). Membranes were exposed to horseradish peroxidase-conjugated secondary antibodies anti-mouse (dilution 1:4000) and anti-rabbit (dilution 1:10000) followed by chemiluminescence (ECL, Amersham Biosciences) detection by autoradiography.

### Confocal Laser Scanning Microscopy (CLSM)

Cells were grown on glass coverslips at the indicated times post infection, they were fixed with 4% paraformaldehyde for 20 min, permeabilized with PBS-0.2% Triton X-100 for 15 min at RT and blocked with PBS-5% BSA for 30 min at RT. The cells were stained with an anti-CD2v polyclonal antibody (diluted 1:750 in PBS-5% BSA), anti-p72 monoclonal antibody (diluted 1/250), anti-vimentin (diluted 1/100), anti-γ-Adaptin monoclonal antibody (α-AP-1) (diluted 1:1000) for 60 min at RT, followed by incubation with an anti-mouse Alexa Fluor-488, anti-rabbit Alexa Fluor-555 or anti-mouse Alexa Fluor-555 for the time indicated. Phalloidin Alexa-647 (diluted 1:100) was used to stain actin microfilaments, and Topro3 (diluted 1:500) and DAPI were used to stain the cell nuclei.

Samples were analyzed by CLSM (Zeiss LSM510 and LSM710) with a 63X oil immersion objective. To analyze CD2-AP-1 co-localization, 3 Z-slices per image from a central plane were collected and deconvolutioned. We used Huygens 3.0 software (Scientific Volume Imaging) for deconvolution and ImageJ (http:/rsb.info.nih.gov/ij) for colocalization graphics. Colocalization was quantified using ImageJ (JACoP) colocalization software and Manders’s Colocalization Coefficients (MCC). Threshold values were determined using automatic threshold (Costes’s automatic threshold).

Images were exported in TIFF format, and their brightness and contrast were optimized with Adobe Photoshop.

### Computational methods

The order/disorder analysis of the cytosolic sequence of the CD2v protein (UNIPROT ID Q89501) was carried out using the IUPred [35] web server in the long and short disorder modes. In both cases, amino acids with scores between 0.5 and 1.0 were regarded as disordered and highlighted (S3 Fig). Similarly, the Anchor web interface [36] was used to determine the disordered regions of the sequence likely to undergo a disorder-to-order transition upon binding to a globular protein partner. The protein sequence was scanned to search for eukaryotic linear motifs (ELMs) using the ELM server [31] with a probability cut-off of 0.1 and the cellular localization filter set to cytosol.

## Results

### CD2v targets viral factories and colocalizes with AP-1 both when ectopically expressed and during ASFV infection

**Fig 1:**
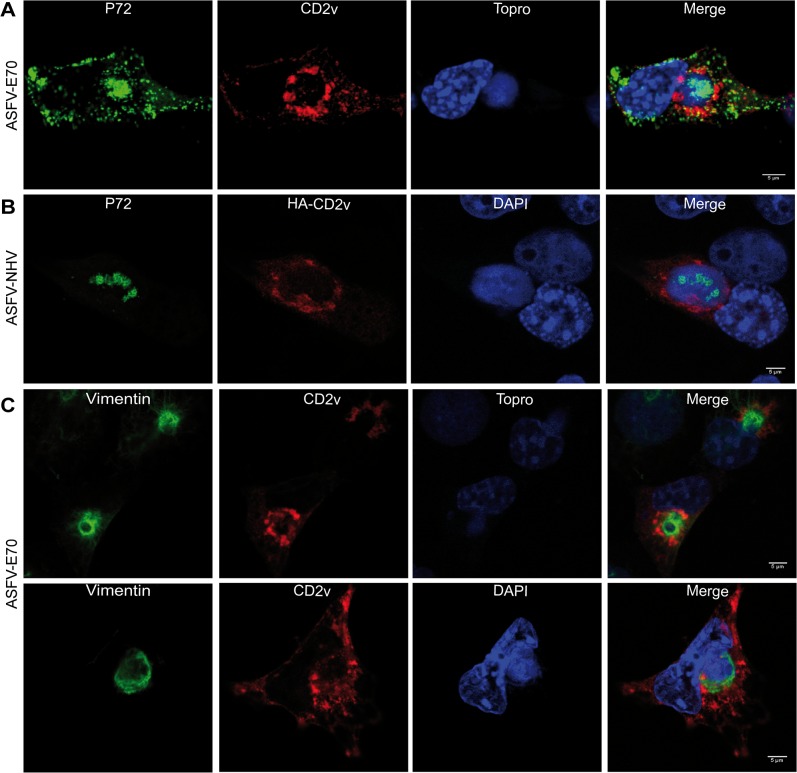
Localization of CD2v around the viral factory during ASFV infection. A: ASFV-E70 infected COS cells were immune stained with α-P72 to detect the viral factory, and with α-CD2v after 16 hpi. B: COS cells were previously transfected with HA-CD2v for 24 hpt, before being infected with the ASFV-NHV strain, which does not express CD2v, for 16 hpi, and immune stained with α-p72 and with α-CD2v. C: ASFV-E70 infected COS cells were immune stained with α-vimentin and α-CD2v after 16 hpi. Images of apical and medial slides are shown in the upper and lower panels respectively.

To explore the function of CD2v during ASFV infection, we first analyzed the distribution pattern of the viral protein in ASFV-infected COS cells with two different strains of ASFV: the virulent E70 (Fig 1A) and the adapted Ba71V strain (Fig A in S1 Fig). As shown in Fig 1A, CD2v localized around the viral factory at 16 hpi, a time by which p72, the major protein of the viral capsid that is usually used to localize the ASFV- factory [37], was distributed into viral factory colocalizing with viral DNA. This distribution of the viral CD2v protein in ASFV infected cells is in agreement with previous results that localized a construct expressing ASFV-Malawi CD2v in Vero cells and infected with ASFV-Ba71v [12]. An alternative experiment was run by transfecting COS cells with a plasmid encoding tagged HA-CD2v, followed by infection with ASFV NH/P68 (NHV), an attenuated strain that does not encode CD2v [15], to analyze the localization of HA-CD2v. As shown in Fig 1B, the product encoded by the plasmid HA-CD2v showed a pattern compatible to that observed during infection, suggesting that the viral protein displays specific localization signals. This specific pattern suggests that CD2v may carry out its function during the infection thereby probably regulating the formation of the viral factory and/or its dynamics. To further investigate this hypothesis, we studied other cellular factors known to be involved in the development of viral factories. Vimentin is an intermediate filament that can be used by several viruses to mediate replication [38–40] and is known to act as a scaffold for the formation of ASFV factories [41,42]. Consequently, we explored whether CD2v would interact with vimentin as a mechanism to target the viral factory. Our results showed that CD2v did not co-localize with vimentin but was clearly identified around the scaffold formed by the intermediate filament (Fig 1C), thus reinforcing the hypothesis of a specific role for CD2v in maintaining the architecture of the viral factory. The distribution pattern of vimentin in ASFV infected cells is compatible which that was previously described [41, 42], even if in our hands, some samples exhibited a more condensed pattern (Fig 1C, upper panel). This difference in vimentin distribution is dependent on the acquired z-slide by CLSM and is also observed when p72 protein distribution was analyzed (Fig B in S1 Fig).

**Fig 2:**
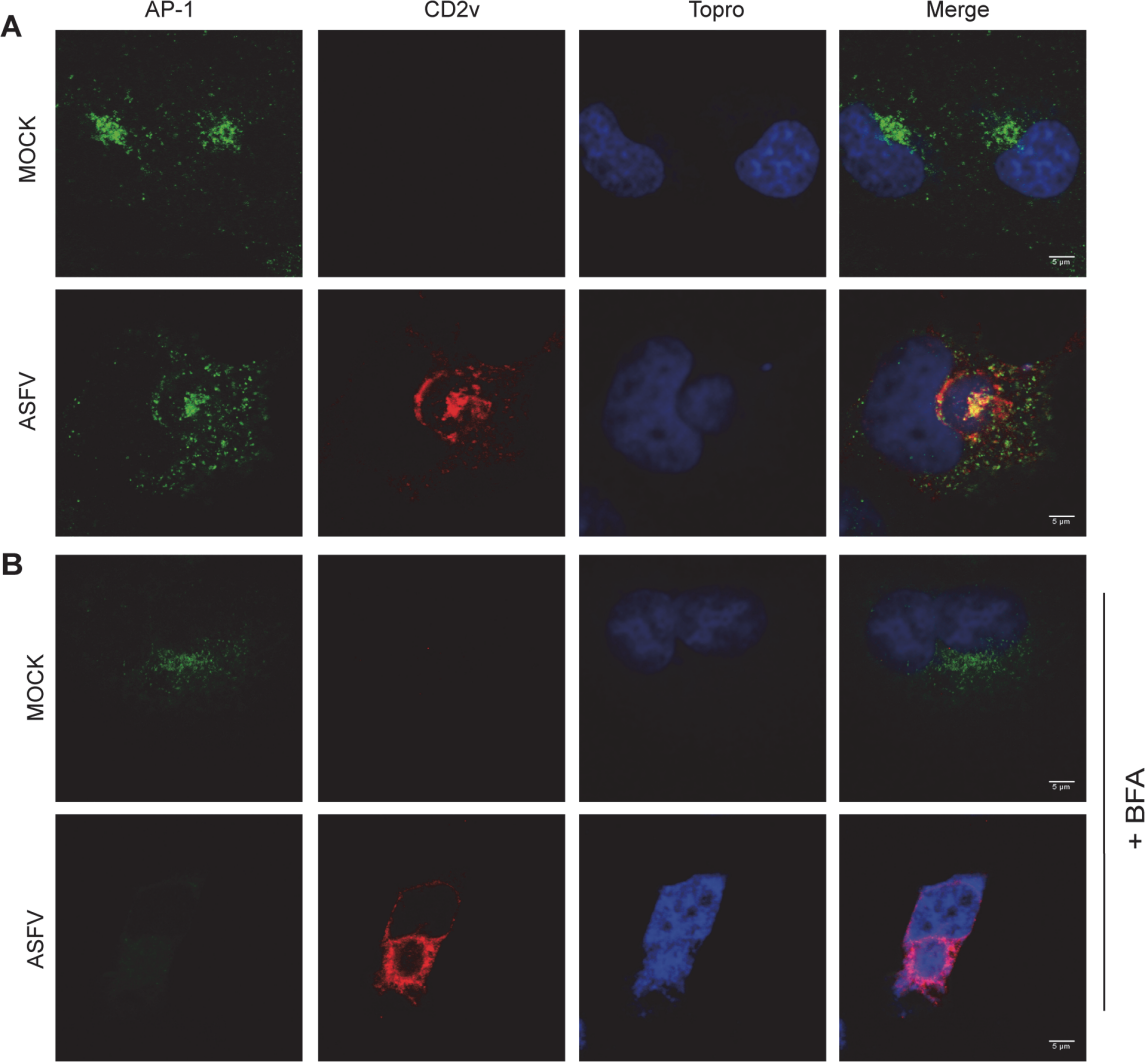
CD2v colocalized with AP-1 in both infected and transfected cells. A: COS cells were MOCK-infected or infected with ASFV-E70 and immune-stained 16 hpi with α-γ-adaptin (AP-1) and α-CD2v. B: COS cells were MOCK-infected or infected with ASFV-E70 for 6h, and then treated with BFA for 3 h. At 9 hpi, cells were immune-stained as in A.

Using ectopically expressed CD2v constructions it has been suggested that the viral protein traffics through the Golgi and ER [12,16]. Since these works do not detect the ASFV protein, but only artificial constructs under the control of the specific viral promoter, it is interesting to analyze the traffic of the native CD2v during the viral infection. To achieve this, and to explore the possibility of CD2v contribution to Golgi and viral factory reorganization, we further characterized cellular markers of the TGN, potential candidates for interaction with the viral protein. One of the major players in Golgi organization is the AP-1 protein complex, which is involved in vesicle traffic between TGN and endosomes and is subverted by several viruses in order to modulate the infection [22,25,27,28,30,43]. Importantly, immune fluorescence assays showed that CD2v colocalized with AP-1 during ASFV-E70 infection in COS cells (Fig 2A) thus reinforcing a putative role for CD2v in Golgi reorganization during ASFV infection and in agreement with previous work showing ectopic CD2v in Golgi compartments [12, 16].

We next investigated whether BFA can disrupt CD2v-AP-1 colocalization. As previously mentioned, BFA leads to the AP-1 delocalization from membranes to cytoplasm [19,44]. Recruitment of AP-1 to the TGN membrane is regulated by Arf1 which, in turn, is regulated by GAPs and GEFs, some of these GEFs families being sensitive to BFA [20]. As shown in Fig 2B, by adding BFA we completely dispersed the AP-1 signal both in uninfected and ASFV-infected COS cells. However, CD2v localization was not affected and remained localized around the viral factory. This result suggests that CD2v targets the viral factory via a specific molecular mechanism that may not involve Arf1 cell regulation. Hence, the localization of CD2v seems independent of the localization of AP-1, indicating that the mechanism of CD2v distribution is different than that used by AP-1 complex, and suggests that the interaction should occur when both proteins reach the TGN.

### AP-1 colocalization depends on the CD2v carboxyl-terminal domain

**Fig 3:**
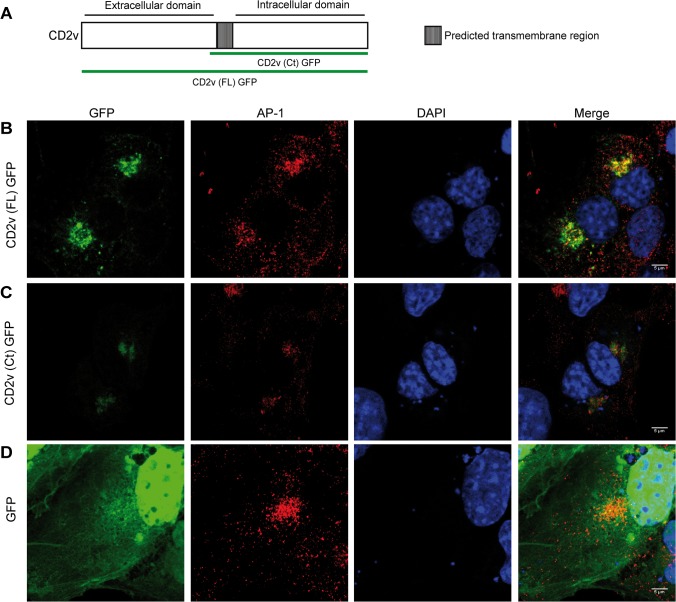
Both full length CD2v and the Ct domain colocalize with AP-1 in COS transfected cells. A: schematic constructs of CD2v-GFP. COS cells were transfected either with the full length CD2v (B), the CD2v Ct domain (C) of GFP fusion proteins or the GFP vector (D). 24 hpt cells were stained with α-γ-adaptin (AP-1) and AP-1 and GFP fusion proteins were observed.

To investigate whether CD2v protein expressed in cells independently of ASFV infection also colocalizes with AP-1, we previously assessed whether the CD2v-GFP construction was recognized by the antibody against CD2v (S2 Fig) when transfected in COS cells. Using this approach, we found that CD2v-GFP clearly colocalized with AP-1 (Fig 3, panel A), further supporting the hypothesis that both that CD2v contains specific localization signals independent of the viral infection and that AP-1 colocalization does not depend on other viral factors.

CD2v is predicted to be a transmembrane protein with two different domains in which the carboxyl terminal (Ct) region is predicted to display localization signals [12]. To explore whether the Ct domain of the protein encodes signals to localize with AP-1, we expressed CD2v-GFP together with a version containing the Ct domain including the predicted transmembrane region (122–402 aa) (CD2v-Ct-GFP) (Fig 3, panel A). Both the full length and the Ct constructions colocalized with AP-1 in COS transfected cells (Fig 3, panels B and C), indicating that signals determining cellular distribution and AP-1 interaction are located in the Ct domain of CD2v. The GFP vector alone was analyzed as negative control (Fig 3, panel D).

### AP-1 interacts with both glycosylated and non-glycosylated forms of CD2v during ASFV infection

To investigate whether the observed colocalization of CD2v and AP-1 could involve an interaction between the two proteins, we conducted a set of immunoprecipitation studies in ASFV-E70-infected COS cells using anti γ-adaptin AP-1 specific antibody. It is interesting to note that CD2v is a glycosylated protein with predicted glycosylation sites located mainly on the Nt domain [11,12], and the second aim of our study was to investigate whether glycosylation of CD2v is a precondition for binding to AP-1.

**Fig 4:**
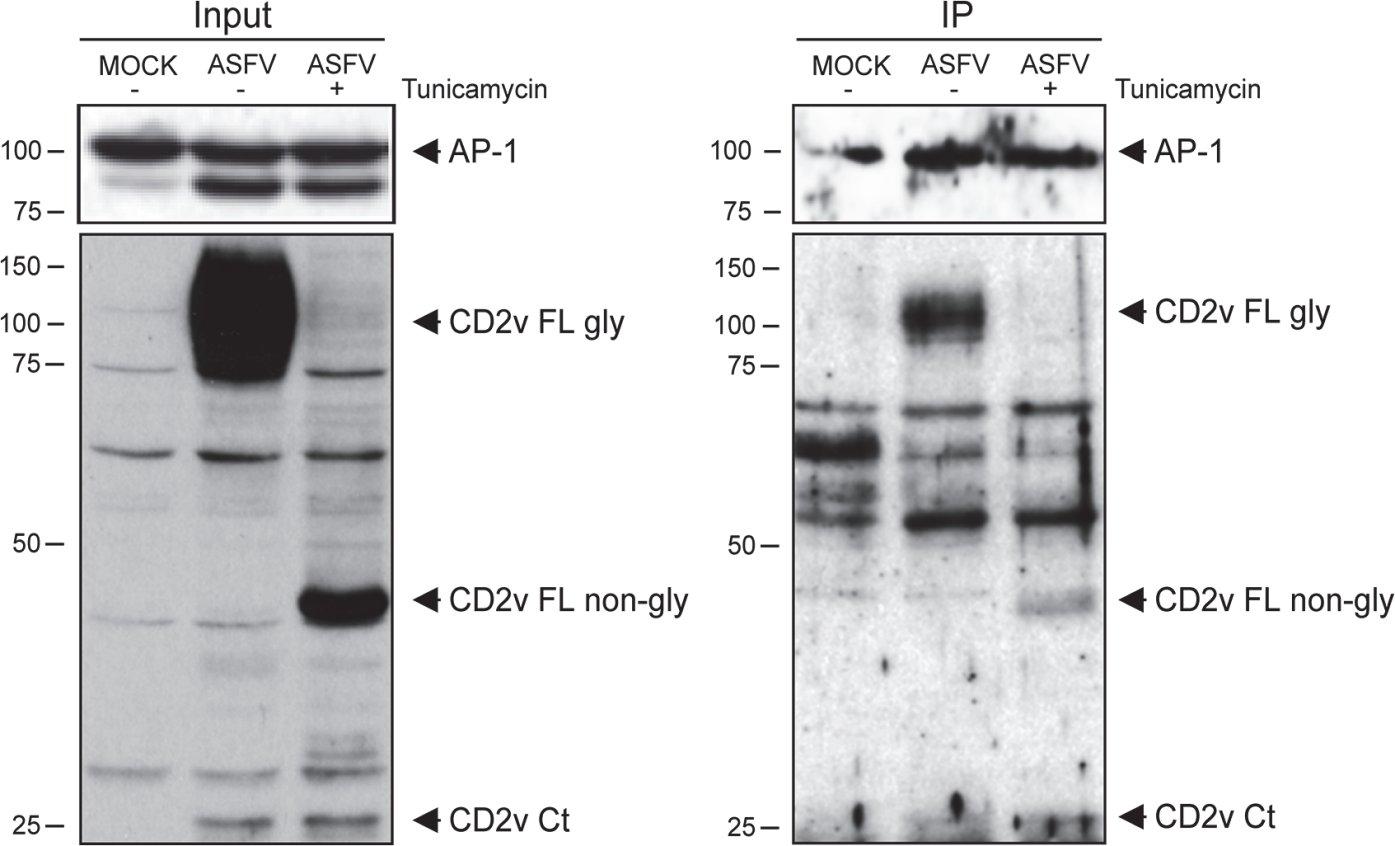
CD2v interacting with AP-1 during ASFV infection. COS cells were MOCK-infected or infected with ASFV-E70 in the presence or not of tunicamycin (5 μg/ml). After 16 hpi, cells were lysed and immune precipitated with α-γ-adaptin (AP-1) as explained in Material and Methods. In ASFV infected cells (left panel, Input) bands corresponding to the glycosylated (110kDa) and non-glycosylated CD2v (42kDa) full length forms of CD2v and the predicted cleaved CD2v Ct domain (26kDa) were observed. In the right panel (IP) the immunoprecipitated and coimmunoprecipitated AP-1 and CD2v are indicated.

COS cells were treated or not with tunicamycin (see Material and Methods) to inhibit glycosylation, and then infected with ASFV-E70. CD2v metabolism includes the glycosylation of a viral polypeptide of 42kDa changing its molecular weight to around 110 kDa. Furthermore, a smaller CD2v peptide of 26kDa is likely to be non-glycosylated during the infection, as its molecular weight remained indentical after tunycamicin treatment (Fig 4, Input) [11, 12]. Infected cells were lysed at 16 hpi and AP-1 was specifically immunoprecipitated with an anti-γ-adaptin antibody from the cleared lysate. Coimmunoprecipitated CD2v was resolved by Western blotting using a specific polyclonal anti-CD2v antibody. As shown in Fig 4, a specific band of 110kDa revealed interaction of AP-1 with the complete CD2v-glycosylated form. Immunoprecipitation of infected cells treated with tunicamycin, a specific band of 42 kDa corresponding to the unglycosylated form of CD2v, was also detected. Unspecific bands are also recognized by the polyclonal anti-CD2v antibody but their potential role in CD2v-AP-1 is discarded since these bands also appeared in the MOCK-infected cells sample. These results indicate, first, that CD2v and AP-1 interacted during ASFV infection, and second, that this interaction was not dependent on glycosylation of CD2v; hence, it could be also speculated that interaction with AP-1 should be an event occurring through a region of CD2v lacking of glycosylation sites. It is also worth noting that an extra band of 26 kDa corresponding to the cleaved cytosolic tail of CD2v, which is not glycosylated, was also observed, suggesting the CD2v-AP-1 interaction could pass through this region.

### Sequence analysis of ASFV-CD2v

To identify the CD2v region that mediates binding to AP-1, using in silico prediction tools, we first searched the Ct sequence for potential interaction sites. Preliminary analysis with IUPred showed that the Ct region of CD2v is most likely disordered (S3 Fig), suggesting that binding to AP-1 is most likely mediated by short linear motifs (SLiMs). These are short functional regions mainly located in disordered regions of the proteins [45] and are involved in multiple cellular processes [46]. Analysis of the Ct region of CD2v for linear motifs using the eukaryotic linear motif resource [31] revealed many potential SLiMs (S1 Table). Among these, we focused on the TRG_LysEND_APsAcLL_1 di-Leu motif localized in the 349–354 region (QNISLI). Surprisingly, this type of motif has also been reported in the HIV Nef protein (UNIPROT ID P04604), an accessory protein that is also involved in virulence enhancement.

### ASFV CD2v-di-Leu motif in a HIV-Nef context supports AP-1 binding

**Fig 5:**
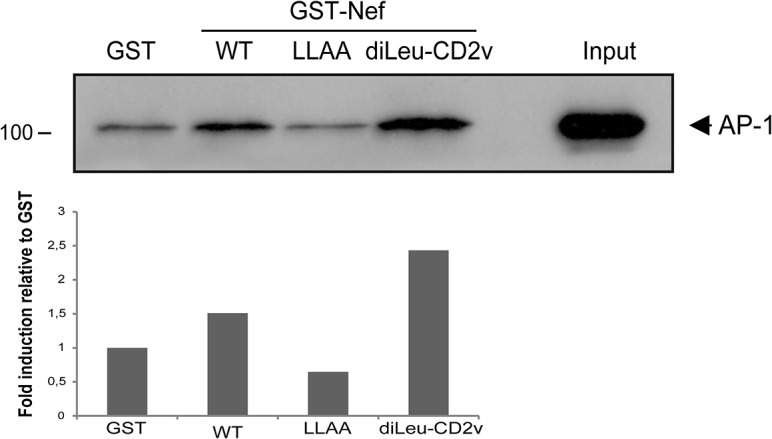
CD2v di-Leu motif is functionally interchangeable with the HIV-Nef di-Leu motif in the AP-1 binding assay. COS cells were lysed and incubated with immobilized GST-Nef-WT, GST-Nef-LLAA, GST-Nef-diLeuCD2v or GST alone and coprecipitated with glutathione-Sepharose beads, separated by 10% SDS-PAGE followed by immunoblotting with an anti-γ-adaptin (AP-1) antibody. Densitometry values of anti-AP-1 bands relative to anti-GST are presented in the graph below. A representative experiment of at least three independent experiments is shown.

Di-Leu motifs have been observed in the cytoplasmic domains of several mammalian and yeast proteins and confer functional activities to these sequences by binding to AP complexes. In particular, this type of motif has been found in the Nef protein family [23] and, interestingly, has also been computationally identified in the Ct sequence of CD2v (S1 Table). It could thus have similar functions, i.e., it is possible that CD2v-AP-1 binding may pass through this motif. To validate this hypothesis, we investigated whether the CD2v di-Leu motif was able to interact with AP-1 complex to complement Nef-AP-1 interaction by substituting the Nef di-Leu motif (ETNSLL) for the CD2v di-Leu motif (QNISLI). Next, we performed a pull-down assay using GST fusion proteins of either the Nef wild type (WT), the deficient mutant Nef-LLAA, or the chimera Nef-di-Leu-CD2v. As shown in Fig 5, the AP-1 complex interacted with both Nef WT and Nef-di-Leu-CD2v but not with the Nef-LLAA mutant, indicating that the CD2v di-Leu motif functionally supported the Nef interaction with AP-1. These data suggest that the CD2v-AP-1 interaction could be mediated by the di-Leu motif we identified.

### CD2v-AP-1 binding is not supported by a di-Leu motif

**Fig 6:**
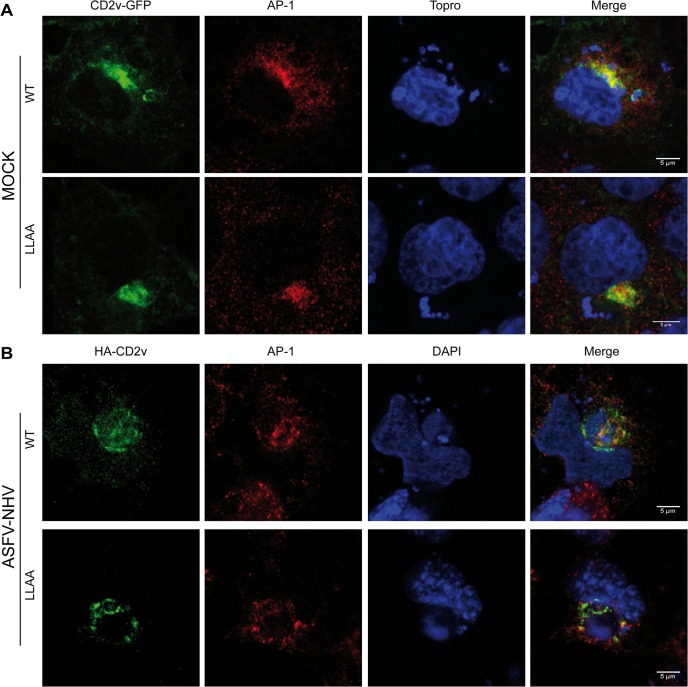
The di-Leu motif is not involved in CD2v-AP-1 co-localization. (A): COS cells were transfected with either wild-type CD2v-GFP or LLAA mutant. 24 hpt, the localization of both CD2v and AP-1 (immune stained with α-γ-adaptin) was examined by confocal microscopy. (B): COS cells were transfected with either wild-type HA-CD2v or LLAA mutant and then infected for 16 h with the ASFV-NHV. CD2v and AP-1 were immune stained with α-CD2v and α-γ-adaptin respectively and examined by confocal microscopy.

Based on these interaction results, we next investigated the CD2v-AP-1 interaction and the involvement of the di-Leu motif identified in CD2v. To this end, we generated the CD2v LLAA mutant, in which the LI of the di-Leu motif (QNISLI) was substituted for AA (QNISAA) in order to disrupt the functionality of the motif. COS cells were transfected with either CD2v-GFP wild-type or with the CD2v-GFP LLAA mutated version and the distribution pattern of the constructs and AP-1 was analyzed. When immunofluorescence experiments were performed, unexpectedly, both CD2v-GFP WT and LLAA colocalized in a similar way with AP-1 (Fig 6A). To analyze these results statistically, we performed quantitative confocal immunofluorescence analysis, in which 10 to 15 cells from each category were randomly chosen and analyzed using ImageJ (JACoP) software and Manders’ colocalization coefficients. M1 and M2 coefficients were 0.82±0.18 and 0.90±0.08 for CD2v-WT and 0.70±0.19 and 0.87±0.11 for CD2v-LLAA respectively, indicating that there were no significant differences in the degree of colocalization. In addition, even when cells were treated with cycloheximide (100 μg/ml) for 1 h to better distinguish potential differences in the subcellular distribution of these molecules, no significant differences were identified (S4 Fig).

To investigate whether potential viral factors could be involved in the distribution of CD2v, we explored the distribution of the products in an infection context. For this purpose, COS cells were transfected with either HA-CD2v WT or HA-CD2v LLAA and then infected with the lacking CD2v strain NHV for 16 h. Interestingly, and confirming our previous results, no differences in sub-cellular distribution patterns were observed, since both HA-CD2v WT and LLAA were found in close association with AP-1 (Fig 6B).

After these results, we finally concluded that neither the sub-cellular distribution nor the AP-1 colocalization was affected by the di-Leu motif on CD2v.

**Fig 7:**
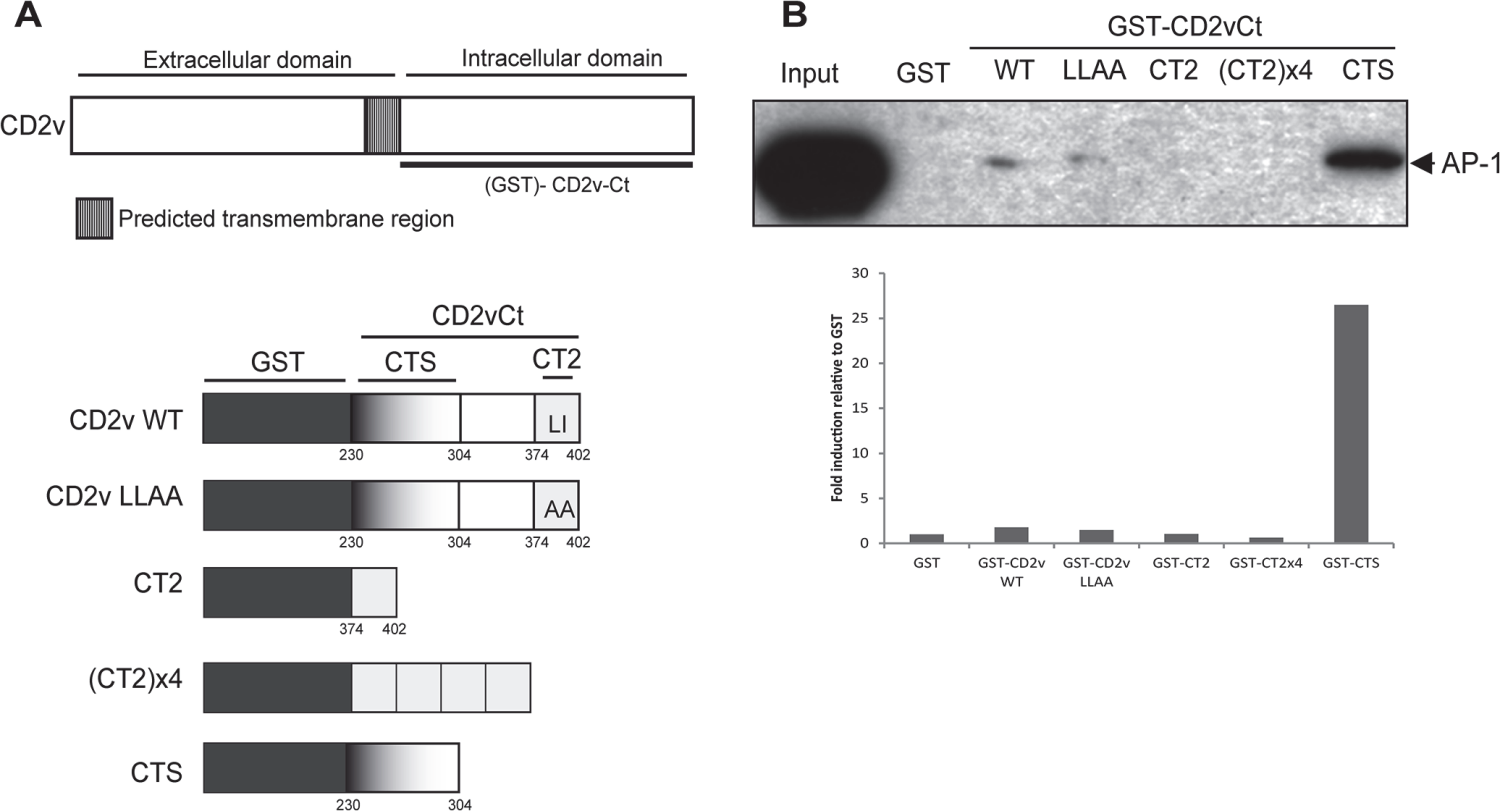
The CD2v-AP-1 interaction is mediated by the Ct domain of CD2v but not by the CD2v di-Leu motif. (A): Structure of GST-fusion proteins of different Ct regions of CD2v: Ct domain of CD2v (240–402 aa), wild-type (CD2v WT), or the di-Leu mutant (CD2v LLAA), 374–402 aa region of CD2v Ct harboring the di-Leu motif in one (CT2) or four copies (CT2x4) and the 240–304 aa region that does not harbor the di-Leu motif (CTS). (B): COS cells were lysed and incubated with immobilized GST-CD2v-WT, GST-CD2v-LLAA, GST-CT2, GST-CT2x4, GST-CTS or GST alone. They were co-precipitated using glutathione-Sepharose beads, separated by 10% SDS-PAGE followed by immunoblotting with an anti-AP-1 monoclonal antibody. Densitometry values of anti-AP-1 bands relative to anti-GST are presented in the graph below. A representative experiment of at least three independent experiments is shown.

Despite the fact the LLAA mutant construction still colocalized with AP-1 as described above, the putative involvement of this motif in the CD2v-AP-1 interaction demonstrated in Fig 4 could not be overlooked. For this investigation, we generated GST-CD2v-Ct recombinant proteins excluding the predicted transmembrane region (230–402 amino acids), containing either the wild-type sequence or the LLAA mutation and assayed them in pull-down assays. In addition, we generated a GST-CD2v Ct encompassing only the last 30 aa (374–402 aa) (CT2) and a 4x tandem repeat of this region to better study the individual role of the di-Leu motif in the interaction with AP-1 (Fig 7A). Unexpectedly, we were unable to detect any substantial differences in AP-1 binding with either the wild-type or the LLAA mutant (Fig 7B and S5 Fig). Interestingly, no interaction was obtained using the isolated di-Leu motif either alone or in the 4x repeat (Fig 7B).

These results clearly show that the CD2v-AP-1 interaction is not sustained by its di-Leu motif but suggest the presence of a non-consensus AP-1 binding domain on the Ct domain of CD2v. To test this possibility, we generated a GST-CD2v-CTS fusion protein that encompasses the first 70 residues (230–304 aa) of its cytosolic region (Fig 7A). Unexpectedly, we found that this region interacted with AP-1 (Fig 7B).

Taken together, these results fully refute the involvement of the di-Leu motif in CD2v-AP-1 binding, raising the possibility that other motifs or regions contained in the 230–304 aa region are responsible for the binding and probably the co-localization of the two proteins. Further studies are planned to identify these potential motifs.

## Discussion

CD2v is an ASFV protein that is predicted to be truncated in several attenuated strains and that is believed to be involved in virulence and infectivity [3]. Nevertheless, virulence in domestic pigs cannot be exclusively attributed to CD2v. In the attenuated NHV strain other genomic differences may contribute for the low virulence phenotype, since when pigs where infected by either the CD2v gene deletion mutant or the wild type virus encoding CD2v all pigs died [13]. However, in the pigs infected with the ΔCD2v mutant a delay in generalization of the infection, in the virus spread and in the onset of the viremia was observed, together with a reduction in virus titters, suggesting that CD2v in some way contributes to the increasing of the virulence.

By the other hand, while CD2v was shown to be involved in disease onset and spreading *in vivo*, unexpectedly, no differences in growth were found *in vitro*, where the Malawi CD2v gene deletion mutant, its revertant and the parental hemadsorbing virus exhibit indistinguishable growth characteristics on primary porcine macrophage cell cultures [13]. Nevertheless, it is important to note that these *in vitro* assays were done at equal initial multiplicity of infection (MOI) from both wild-type and ΔCD2v virus. Since titration and initial MOIs are calculated based on viral ability to infect, the possible differences in productive, infectious virions, may be minimized. Further experiments are planned to avoid this technical drawback.

Here, we showed that viral E70 CD2v was localized around the viral factory at different times after infection, as well as when ectopically expressed [12,16]. In transfected cells, CD2v was distributed in TGN cellular compartments compatible with those occupied by the viral factory during viral infection. Furthermore, by using cells infected with an attenuated strain deficient for CD2v expression (NHV) and after transfection with several CD2v constructions, ectopically CD2v was clearly localized around the factory similar to the way CD2v localizes during E70 infection. These results indicate that the sub-cellular distribution of CD2v depends on specific peptide localization signals, targets the viral factory and/ or related cellular compartments, and suggest that the protein may be involved in the architecture of the viral factory.

Interestingly, we observed that the clathrin adaptor complex AP-1 colocalized with CD2v around the viral factory thus strongly suggesting protein-protein interaction events. AP-1 has been shown to interact with several viral proteins including HIV-Nef and BPV-E6 [22,28]. The interaction between viral proteins and AP-1 has been proposed as a mechanism to subvert host cellular trafficking to ensure and optimize viral morphogenesis and viral egree while allowing immune evasion of the infected cell [25,27,28,43].

AP-1 localization in TGN membranes depends on the previous Arf1 localization, which in turn depends on a GTP-GDP cycle controlled by specific GAPs and GEFs. BFA specifically inhibits three GEFs: GBF1, BIG1 and BIG2 [20]. The regulation of these components is crucial for morphogenesis in viruses like Coxsackievirus B3, in which RNA replication depends on GBF1 activity [47]. It is noteworthy that during BPV infection, E6 protein competes with AP-1 for the same membrane binding locations, suggesting a common factor may be involved in the localization of the two proteins [28]. Finally, HIV-Nef binds to AP-1 thereby stabilizing the binding of AP-1 to the membranes through an Arf1-independent mechanism, since BFA treatment does not disperse to either AP-1 or Nef [24]. It is likely that CD2v acts in a different way, since BFA was able to interfere with binding to AP-1 dispersing AP-1, while CD2v remained attached to the TGN membrane around viral factories.

These results show that the localization of CD2v does not depend on the localization of AP-1, suggesting that the interaction could occur as soon as both proteins reach this compartment. In particular, we suggest that either CD2v interacts with AP-1 to specifically localize in the TGN around the viral factory, or hijacks the AP-1 dependent machinery to localize in TGN membranes.

The interaction between CD2v and AP-1 occurs independently of the glycosylation of the viral protein, since non-glycosylated CD2v interacted with AP-1. CD2vCt-GFP remained colocalized with AP-1 in COS transfected cells and CD2v-Ct was able to interact with AP-1 in a pull-down assay, in agreement with a differentiated function for the Nt and Ct fragments of CD2v, and reinforcing the hypothesis of a role for the Ct domain in this interaction.

Two signals of cargo proteins recognized by the adaptor protein complexes AP-1, AP-2 and AP-3 have been reported: the tyrosine based motif (YxxΦ) and the di-Leu motif ([DE]xxxL[LI]) [18]. Although there is no Tyr motif in the Ct sequence of CD2v, a di-Leu motif was found at the end of the Ct motif of CD2v (QNISLI). This di-Leu motif was complementary to the di-Leu motif described in Nef, and was further able to rescue the binding to AP-1 in a Nef- AP-1 pull-down assay.

However, we did not observe any differences in colocalization between CD2v and AP-1 by a mutation of the di-Leu motif (LLAA mutant) in immunofluorescence assays, while this mutant was still able to bind AP-1 in pull-down assays. In addition, we identified the 240–304 aa region in the CD2v (CTS), spreading out from the di-Leu motif, as being responsible for binding with AP-1, ruling out the involvement of the CD2v di-Leu motif. The question still remains whether during ASFV infection, the di-Leu motif we identified plays another role in CD2v function, i.e., different than AP-1 binding.

The possibility that other motifs in CD2v involved in cargo recognition may be responsible for this binding is an attractive hypothesis that could be applicable not only in ASFV biology, but also in AP-1 function and cellular traffic. Another possibility is that the interaction between CD2v and AP-1 may not be direct but mediated by another protein or protein complex. A possible candidate for this mediation is clathrin, since clathrin is a protein that binds to AP-1 to form the coated pits, and CD2v harbors a potential motif for clathrin binding (170-LIHVD-174). However, preliminary results failed to detect clathrin in the CD2v-AP-1 complex.

One of the most important consequences of our work regarding ASFV virulence is the physiological implications of the binding of CD2v and AP-1. We hypothesized that the binding of CD2v to AP-1 could play a role in restructuring cell traffic that may affect the architecture of the viral factory, since CD2v surrounded it during infection. However, the presence of BFA (which delocalized AP-1 but not CD2v), does not prevent the formation of viral factories. In addition, during infection with the attenuated NHV strain, which does not encode CD2v, viral factories were still observed, although it is difficult to be sure they were fully functional.

In herpes virus infection, final envelopment occurs in TGN membranes in vesicles containing AP-1 [30], and AP-1 has been related in viral egress [29]. AP-1 also appears to be involved in the viral egress of several flaviviruses [43]. ASFV might form a similar pathway for egression involving AP-1 and consequently CD2v. In fact, ASFV infection causes Golgi dispersion and the impairment of exocytosis machinery [48], and it is an attractive hypothesis that CD2v-AP-1 interaction may play a role in these events, with consequences for virulence and immune escape. The HIV-Nef protein is able to increase virulence and pathogenesis and one of the actors used for Nef to revert the cell physiology and increase pathogenicity is in fact AP-1 [25,27]. Although, as we have demonstrated, the mechanism of Nef-AP-1 binding differs from that of CD2v, since the di-Leu motif is not involved and the binding is not stabilized in the membrane as described for Nef [22,24], the consequences of AP-1 binding may be similar in terms of immune evasion. Nef binds to MHC-I thereby promoting its binding to AP-1 through a Tyr motif [27,49]. As a consequence, MHC-I (A and B, but not C) is sequestered in the cytoplasm and the infected cell is able to escape the control of CTL and NK cells. Advances in our understanding of the mechanism of localization and binding between CD2v and AP-1 may help elucidate these and other aspects linked to immune evasion and infectivity likely depending on CD2v during ASFV infection. The mapping of the interaction could also help develop new antivirals and diagnostic tools.

## Supporting Information

S1 Fig Localization of CD2v (A) and vimentin (B) around the viral factory in ASFV infected cells. (A): CD2v localized around the viral factory in ASFV-Ba71v Vero infected cell. (B): Vimentin localization around the viral factory in ASFV-E70 COS infected cell in an apical (upper panel) and medial slide.
(TIF)

S2 Fig CD2v-GFP is recognized by the polyclonal anti-CD2v antibody. COS cells expressing CD2v-GFP construct, stained with 1:750 diluted anti- CD2v antibody.
(TIF)

S3 Fig In silico analysis of the CD2v-Ct domain. Regions predicted to be disordered are highlighted in red, while disordered regions predicted to become ordered upon interaction with a globular protein are in yellow. Di-Leu predicted SLiM is given above the shaded boxes that indicate the corresponding sequence stretches.
(TIF)

S4 Fig Colocalization of AP-1 with CD2v WT and LLAA mutant in GFP transfected cells pretreated with cycloheximide. CD2v-GFP WT and LLAA mutant COS cells transfected cells, were treated with cycloheximide (100 μg/ml) during 1h before the staining for immunofluorescence.
(TIF)

S5 Fig The di-Leu motif of CD2v is not responsible for AP-1 binding. COS cells were lysed and incubated with immobilized GST-CD2v-WT, GST-CD2v-LLAA, GST-CT2 nor GST alone. They were co-precipitated using glutathione-Sepharose beads, separated by 10% SDS-PAGE followed by immunoblotting with an anti-AP-1 monoclonal antibody. Densitometry values of anti-AP-1 bands relative to anti-GST are presented in the graph below. A representative experiment of at least two independent experiments is shown.
(TIF)

S1 Table Summary of predicted short linear motifs found in the cytosolic region of CD2v [226–402]. (PDF)
