# Supplementary figures and images for: CD2v Interacts with Adaptor Protein AP-1 during African Swine Fever Infection
Source: PLoS One. 2015 Apr 27;10(4):e0123714. doi: 10.1371/journal.pone.0123714 (PMC4411086; doi:10.1371/journal.pone.0123714)

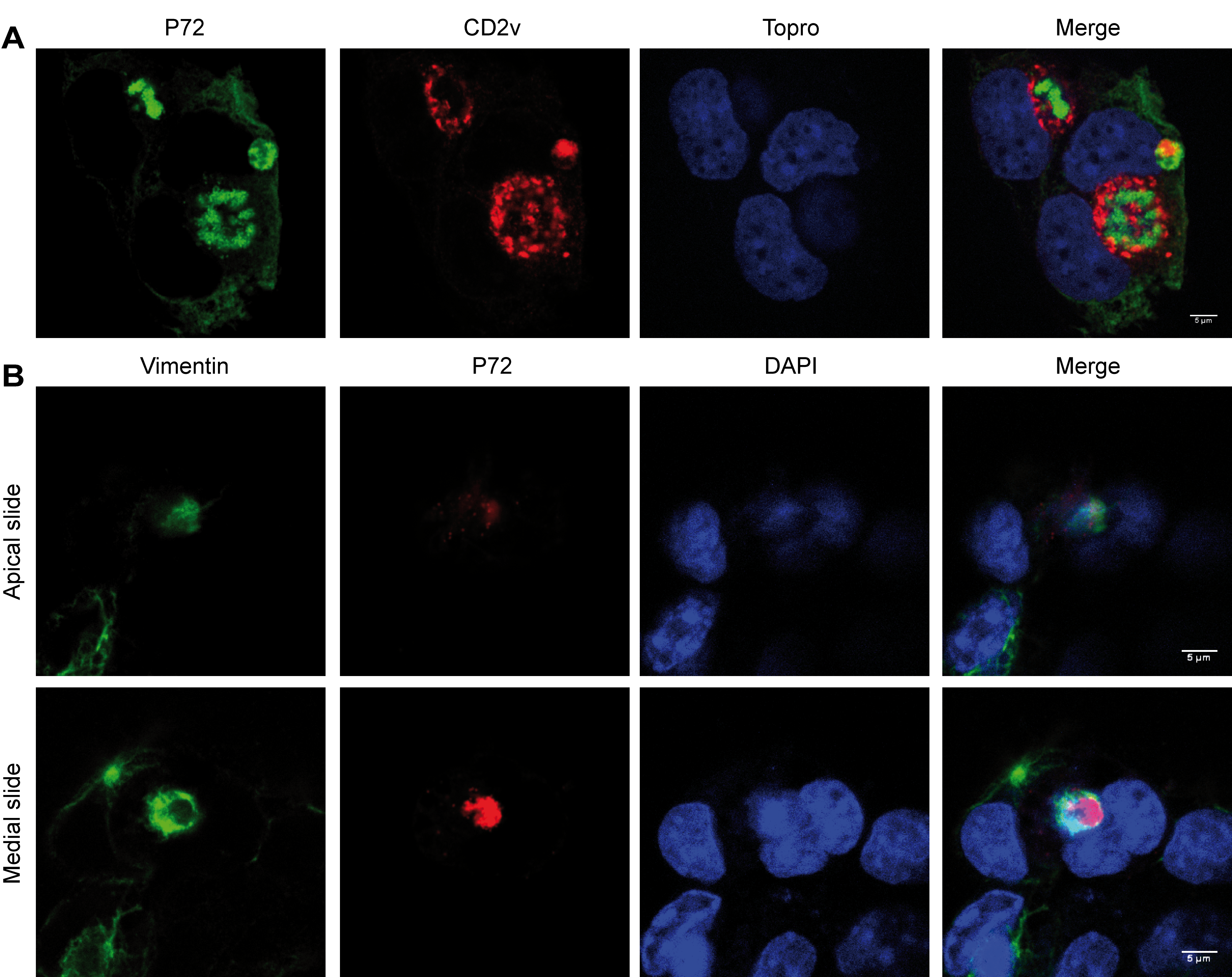

Supplement: S1 Fig — (A): CD2v localized around the viral factory in ASFV-Ba71v Vero infected cell. (B): Vimentin localization around the viral factory in ASFV-E70 COS infected cell in an apical (upper panel) and medial slide. (TIF) [file pone.0123714.s001.tif]

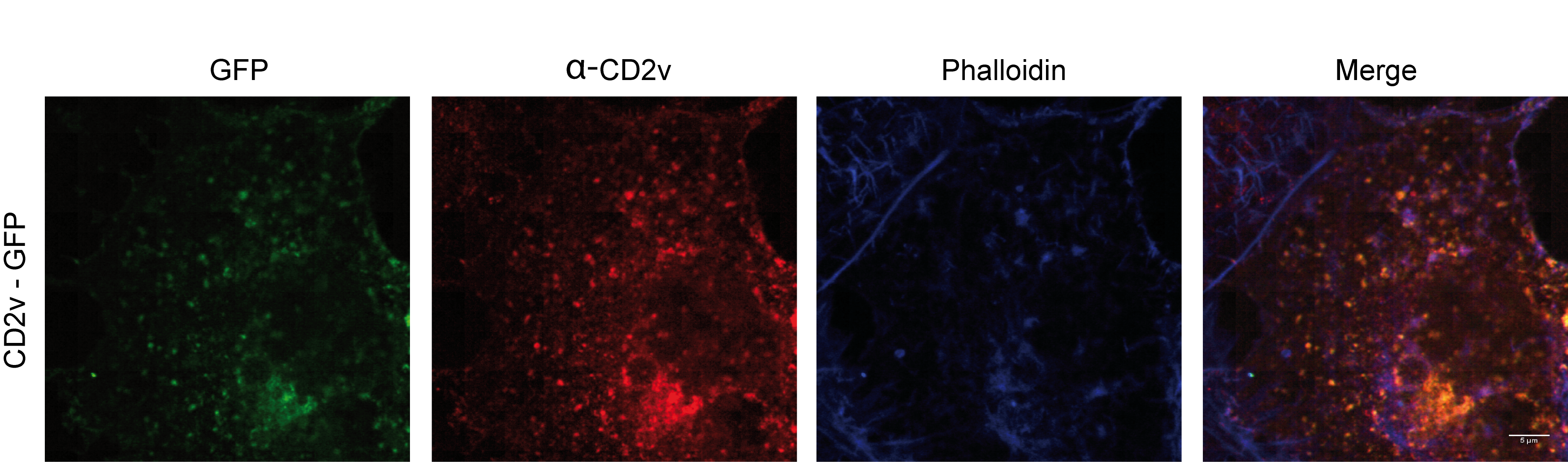

Supplement: S2 Fig — COS cells expressing CD2v-GFP construct, stained with 1:750 diluted anti- CD2v antibody. (TIF) [file pone.0123714.s002.tif]

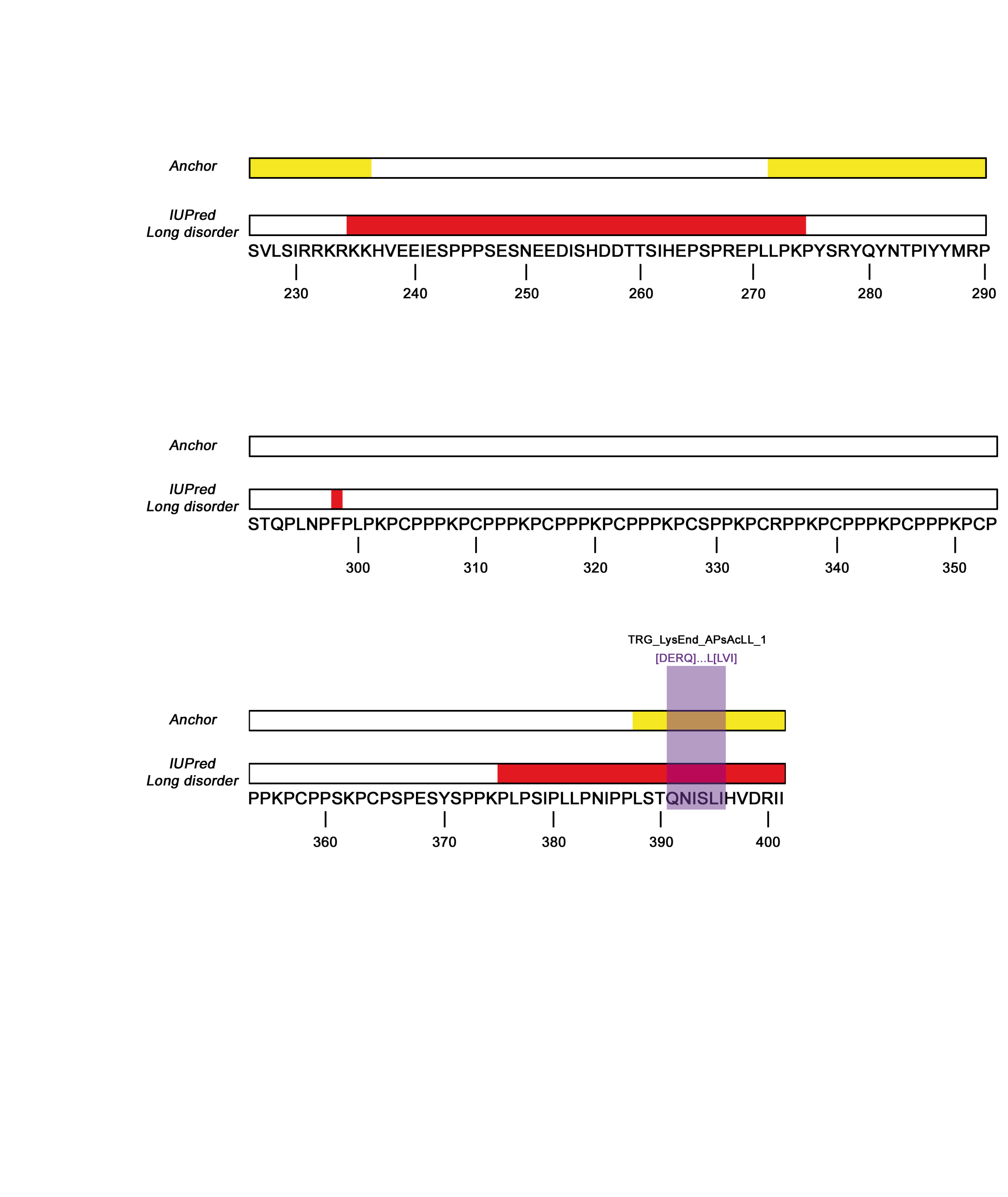

Supplement: S3 Fig — Regions predicted to be disordered are highlighted in red, while disordered regions predicted to become ordered upon interaction with a globular protein are in yellow. Di-Leu predicted SLiM is given above the shaded boxes that indicate the corresponding sequence stretches. (TIF) [file pone.0123714.s003.tif]

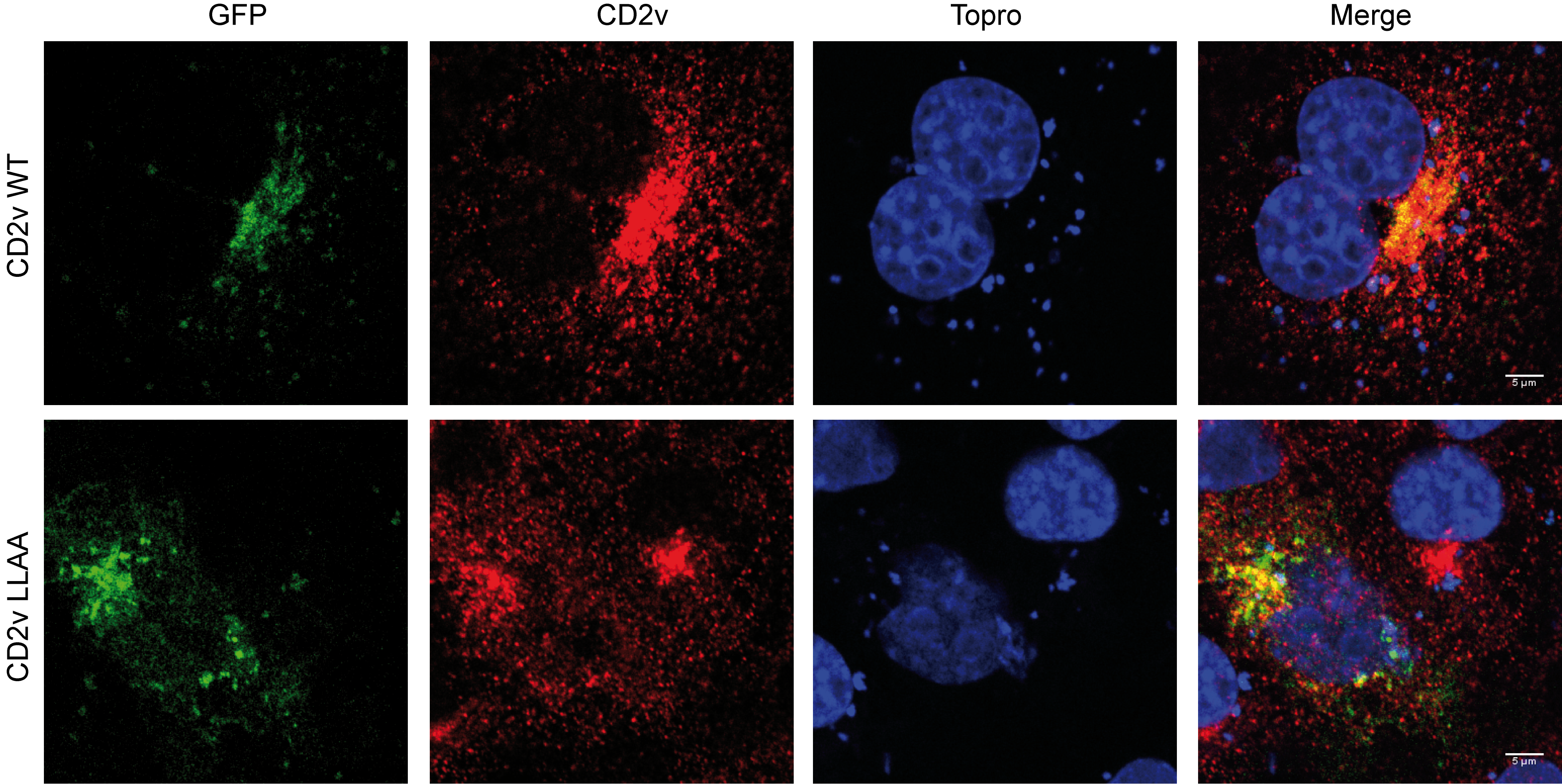

Supplement: S4 Fig — CD2v-GFP WT and LLAA mutant COS cells transfected cells, were treated with cycloheximide (100 μg/ml) during 1h before the staining for immunofluorescence. (TIF) [file pone.0123714.s004.tif]

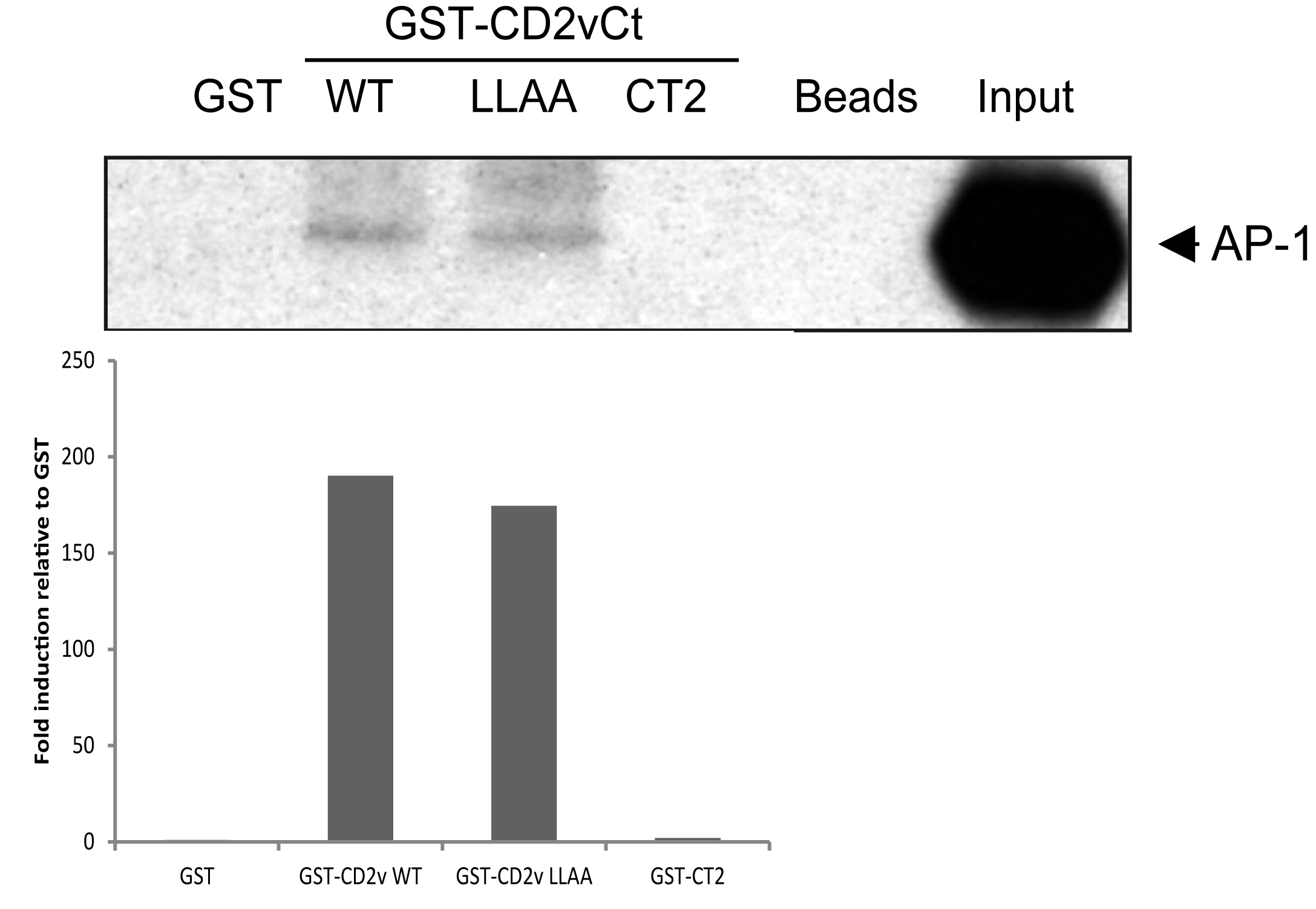

Supplement: S5 Fig — COS cells were lysed and incubated with immobilized GST-CD2v-WT, GST-CD2v-LLAA, GST-CT2 nor GST alone. They were co-precipitated using glutathione-Sepharose beads, separated by 10% SDS-PAGE followed by immunoblotting with an anti-AP-1 monoclonal antibody. Densitometry values of anti-AP-1 bands relative to anti-GST are presented in the graph below. A representative experiment of at least two independent experiments is shown. (TIF) [file pone.0123714.s005.tif]
