# Supplementary material for: CD2v Interacts with Adaptor Protein AP-1 during African Swine Fever Infection
Source: PLoS One. 2015 Apr 27;10(4):e0123714. doi: 10.1371/journal.pone.0123714 (PMC4411086; doi:10.1371/journal.pone.0123714)
Supplement: S1 Table — (PDF) [file pone.0123714.s006.pdf]

Table S1. Summary of predicted short linear motifs found in the cytosolic region of CD2v [226-402].

Name of SLiM	Matched sequence		Pattern	Probability
	Amino acids	Range		
DOC_CKS1_1	YNTPIY	281-286	[MPVLIFWYQ].(T)P..	0.0020
DOC_CYCLIN_1	KPLP	374-377	[RK].L.[28][FYLIVMP]	0.0053
DOC_WW_Pin1_4	EIESPP<br>HEPSPR<br>QYNTPI<br>KPCSPP<br>PCPSPE<br>ESYSPP	240-245<br>263-268<br>280-285<br>326-331<br>363-368<br>348-373	...([ST])P.	0.0154
LIG_14-3-3_1	RPSTQP	289-294	R.[^P]([ST])[^P]P	0.0008
LIG_BIR_II_1	SVLS	226-229	^M{0,1}[AS]...	0.0003
LIG_Clathr_ClatBox_1	LIHVD	395-399	L[IVLMF].[IVLMF][DE]	0.0003
LIG_EH_1	LNPFP	295-299	.NPF.	0.0001
LIG_SH3_1	KPLPSIP	374-380	[RKY]..P..P	0.0012
LIG_SH3_2	PCPPPK<br>PCPPPK<br>PCPPPK<br>PCPPPK<br>PCSPPK<br>PPKPCR<br>PCRPPK<br>PCPPPK<br>PCPPPK<br>PCPPPK<br>PCPPSK	303-308<br>309-314<br>315-320<br>321-326<br>327-332<br>330-335<br>333-338<br>339-344<br>345-350<br>351-356<br>357-362	P..P.[KR]	0.0011

LIG_SH3_3	EPSPREP<br>PREPLLP<br>STQPLNP<br>LPKPCPP<br>PCPPPKP<br>PPKPCPP<br>PCPPPKP<br>PPKPCPP<br>PCPPPKP<br>PPKPCPP<br>PCPPPKP<br>PPKPCSP<br>PCSPPKP<br>PPKPCRP<br>PCRPPKP<br>PPKPCPP<br>PCPPPKP<br>PPKPCPP<br>PCPPPKP<br>PPKPCPP<br>PCPPPKP<br>PPKPCPP<br>PCPPSKP<br>SYSPPKP<br>KPLPSIP<br>PSIPLLP<br>PLLPNIP	264-270<br>267-273<br>291-297<br>300-306<br>303-309<br>306-312<br>309-315<br>312-318<br>315-321<br>318-324<br>321-327<br>324-330<br>327-333<br>330-336<br>333-339<br>336-342<br>339-345<br>342-348<br>345-351<br>348-354<br>351-357<br>354-360<br>357-363<br>369-375<br>374-380<br>377-383<br>380-386	...[PV]..P	0.0132
LIG_TRAF2_1	SNEE	249-252	[PSAT].[QE]E	0.0043
LIG_TRAF6	PPPSESN<br>EE	244-252		0.0017

MOD_CDK_1	KPCSPPK<br>ESYSPPK	326-332<br>368-374	...([ST])P.[KR]	0.0019
MOD_CK1_1	SVLSIRR<br>SPESYSP	226-232<br>366-372	S..([ST])...	0.0170
MOD_CK2_1	PSESNEE<br>DTTSIHE<br>HEPSPRE	246-252<br>258-254<br>263-269	...([ST])..E	0.0145
MOD_GSK3_1	EIESPPPS<br>EDISHDD<br>T<br>PLSTQNI<br>S	240-247<br>252-259<br>387-394	...([ST])...[ST]	0.0268
MOD_PKA_2	MRPSTQ<br>P	288-294	.R.([ST])[^P]..	0.0095
MOD_ProDKin_1	EIESPPP<br>HEPSPRE<br>QYNTPIY<br>KPCSPPK<br>PCPSPES<br>ESYSPPK	240-246<br>263-269<br>280-286<br>326-332<br>363-369<br>368-374	...([ST])P..	0.0154
TRG_ER_diArg_1	IRR<br>RRKR	230-232<br>231-234	([LIVMFYWPR]R[^YFWDE]{0,1<br>}R)|(R[^YFWDE]{0,1}R[LIVMF<br>YWPR])	0.0054
TRG_LysEnd_APsAc<br>LL_1	**QNISLI**	**391-396**	**[DERQ]...L[LVI]**	**0.0028**

Predicted short linear motifs found in the cytosolic region of CD2v (226-402 aa, where the di-Leu motif (TRG_LysEnd_APsAcLL_1) is bold outline.
